# A Qualitative Systematic Review of Access to Substance Use Disorder Care in the United States Criminal Justice System

**DOI:** 10.3390/ijerph191912647

**Published:** 2022-10-03

**Authors:** Rachel E. Barenie, Alina Cernasev, Hilary Jasmin, Phillip Knight, Marie Chisholm-Burns

**Affiliations:** 1 Department of Clinical Pharmacy and Translational Science, College of Pharmacy, University of Tennessee Health Science Center, Nashville, TN 37211, USA; 2 Department of Clinical Pharmacy and Translational Science, Health Sciences Library, College of Pharmacy, University of Tennessee Health Science Center, Memphis, TN 38163, USA; 3 School of Medicine, Oregon Health and Science University, Portland, OR 97239, USA

**Keywords:** substance use disorder, criminal justice system, treatment

## Abstract

Background: The majority of patients with a substance use disorder (SUD) in the United States do not receive evidence-based treatment. Research has also demonstrated challenges to accessing SUD care in the US criminal justice system. We conducted a systematic review of access to SUD care in the US criminal justice system. Methods: We searched for comprehensive qualitative studies in multiple databases through April 2021, and two researchers reviewed 6858 studies using pre-selected inclusion criteria. Once eligibility was determined, themes were extracted from the data. This review provides a thematic overview of the US qualitative studies to inform future research-based interventions. This review was conducted in compliance with the Preferred Reporting Items for Systematic reviews and Meta-Analyses (PRISMA). Results: There were 6858 unique abstract results identified for review, and seven qualitative studies met the inclusion criteria. Two themes were identified from these results: (1) managing withdrawal from medication-assisted treatment, and (2) facilitators and barriers to treatment programs in the criminal justice system. Conclusions: Qualitative research evaluating access to SUD care in the US criminal justice system varied, with some interventions reported not rooted in evidence-based medicine. An opportunity may exist to develop best practices to ensure evidence-based treatment for SUDs is delivered to patients who need it in the US criminal justice system.

## 1. Introduction

Access to evidence-based care for the treatment of substance use disorders (SUD) remains a challenge in the United States (US). In 2020, approximately 40 million Americans had a SUD involving either drugs or alcohol, which is approximately seven percent of the US population. Of those, only 2.6 million reported receiving any treatment, which is approximately 7% [1]. There are several factors that contribute to this disparity, such as stigma, a complex regulatory system to access treatment, lack of knowledge about available treatment options, and more. These data, however, only reflect that of the general population in the US—it does not account for persons in the US criminal justice system.

Considering the US has the largest incarceration rate in the world, it is estimated that 65% of persons in the US prison system meet the criteria for a SUD [2,3]. This is likely an underestimate of all the persons involved with the criminal justice system that may be candidates for treatment since it does not include persons in county jails, on probation, and more [4,5]. Advocates have identified access to SUD care while incarcerated as a serious issue here in the US, even successfully arguing that withholding treatment is tantamount to cruel and unusual punishment in violation of the eighth amendment of the US Constitution along with other federal and state laws [6]. Some have even posited that this failure to provide care in the criminal justice system is another factor that perpetuates the drug overdose epidemic in the US [7].

A body of research is emerging on this topic for specific types of SUDs, such as opioid use disorder, that evaluate interventions that may increase treatment retention and reduce morbidity and mortality for persons in prison. For example, one study found disbelief in SUD treatment may hinder the ability of prisoners to actually receive treatment [8]. Another study found cooperation between criminal justice systems and healthcare systems can support persons in continuing their existing SUD treatment regimen [9]. Other research has shown that naloxone kits in prisons can help prevent overdose deaths [10]. Research in this area is critical to improve access to evidence-based treatment in the US criminal justice system.

To date, little qualitative research exists comparing interventions that support access to care for all types of SUDs across the entire criminal justice system, rather than focusing on specific types of SUDs or pockets of the system. We aimed to identify promising initiatives implemented in the criminal justice system that may best support access to care for people living with SUD in the US.

## 2. Methods

This systematic review was conducted to identify studies focused on persons with SUDs that are involved with the criminal justice system in the US and their access to care for their disorder. This review was conducted in compliance with the Preferred Reporting Items for Systematic reviews and Meta-Analyses (PRISMA). PROSPERO ID number is: CRD42022311748.

**Figure 1:**
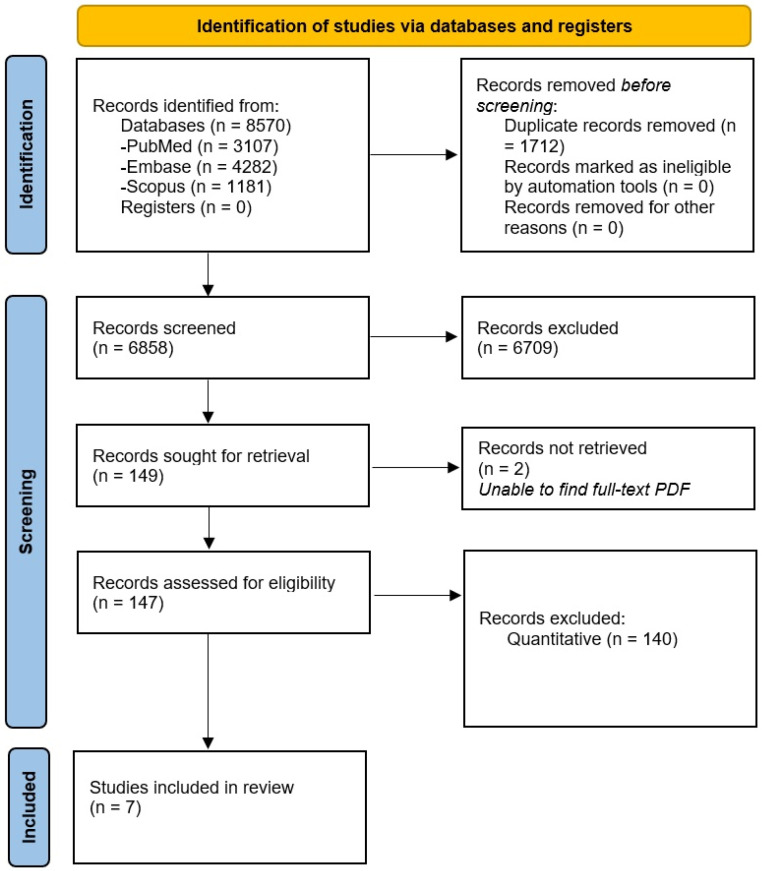
PRISMA Diagram.

Three electronic databases (PubMed, Scopus, and Embase) were searched in April 2021 using a combination of keywords, Medical Subject Headings (MeSH), and/or Emtree subject headings. Search terms were harvested from three primary concepts: drugs of abuse, the justice system and its accompanying language, and healthcare accessibility and services (See Appendix A for full search strategy.) Results from the initial search were imported into EndNote, version 20 (Clarivate, Philadelphia, PA, USA). The unique records were then imported into Rayyan QCRI (Qatar Computing Research Institute, Doha, Qatar), an online platform designed to expedite the screening process. After initial importation, the duplicate records were removed and the remaining abstracts were reviewed by two reviewers independently to make a preliminary eligibility determination. After the preliminary review, a full-text review was conducted of the remaining articles to determine their eligibility into the systematic literature review. The PRISMA Flow Diagram illustrates this process (Figure 1).

### 2.1. Inclusion and Exclusion Criteria

Eligible studies were included if they generally pertained to initiatives in the criminal justice system to support persons with SUDs’ access to care. We focused on US qualitative studies because they illuminate in-depth information on socio-cultural, behavior, and medication experiences perceived by the incarcerated individuals. Furthermore, the qualitative studies facilitate the emergence of common themes across a population [10].

Behavioral, medication, and other types of initiatives aimed to improve care for persons with any type of SUD (e.g., opioid use disorder, alcohol use disorder, stimulant use disorder, nicotine use disorder) were included so long as the initiative took place while the person was involved with the US criminal justice system. Only studies conducted in the US on or after 1970 were included. This was due to the uniqueness of the US criminal justice system, and time period aligns with the passage of the Federal Comprehensive Drug Abuse Prevention and Control Act of 1970, which created the regulatory framework that largely still remains in effect today to govern care for patients with opioid use disorder [11].

Studies were excluded when initiatives did not pertain to patients with SUDs, did not occur in the criminal justice system, or occurred outside the US. Records that were news articles, reports, conference abstracts, court cases, meta-analysis, systematic literature reviews, research monographs, commentaries, research protocols, patent agreements, and policy papers (both in the US and abroad) were excluded. Additional results were excluded that: included historical narratives past events; pertained to the specific pharmacology of a certain substance; drug trafficking; laboratory or toxicology testing, cost effectiveness analyses; or other results with a primary focus on other disease states, such as tuberculosis, human immunodeficiency virus, coronavirus, or serious mental illness. Additionally, studies that pertained to minors or adolescents in the criminal justice system were excluded.

### 2.2. Qualitative Data Analysis

This qualitative systematic literature review aimed to capture new understandings of previously studied concepts and explored the participants’ experiences with the US criminal justice system. Two researchers independently abstracted the included qualitative studies that contained the following categories: author, year of publication, number of interviews or number of focus groups, demographics of participants, treatment received, name of medications, and presented themes [12]. The information extracted from each study was entered into a table to provide an overview for a Thematic Analysis of the articles (Table 1). Both researchers independently summarized each study, discussed, met and reached consensus for the Thematic Analysis [11]. 

## 3. Results

There were a total of 8570 abstract results identified. Of those, 1712 were duplicates and subsequently deleted. A total of 6858 abstract results remained for screening, and 149 abstract results were included for full-text screening to determine their eligibility for inclusion in the review. Two of the 149 could not be retrieved after exhaustive searching for a full-text PDF. Of those 147, seven studies were included in this qualitative systematic literature review and two themes were identified based on Thematic Analysis: (1) managing withdrawal from medication-assisted treatment (MAT), and (2) facilitators and barriers to treatment programs in the criminal justice system.

### 3.1. Managing Withdrawal from Medication-Assistant Treatment

Four of the seven articles have a specific focus on how the withdrawal from MAT programs causes severe disruptions for the incarcerated participants. For example, Mitchell et al., 2011 Thematic Analysis showed a distinct dichotomy in the participants’ responses to the interview questions. Some major themes discussed the significance of withdrawal in subjects dependent on heroin or methadone when they are incarcerated [18]. Other themes revealed that study participants perceived their imprisonment as a restraint from using these drugs. Furthermore, the study highlighted that participants receiving methadone during their time in prison provided an opportunity to abstain from drugs.

Aronowitz et al., 2016 focused on the participants’ perceptions of prisoners whose MAT was tapered or stopped during their time in prison [13]. The Thematic Analysis revealed traumatic experiences described by the participants due to the taper regimens or sudden discontinuation of the MAT. The participants also shared about their difficult withdrawal experiences by highlighting various physical symptoms they suffered. These withdrawal symptoms were reported by both groups of patients—tapering patient group and the group stopping MAT. Furthermore, this study emphasized the need for programs to focus on improving physical and mental health outcomes for patients with opioid use disorder and in prison.

The clinical trial conducted by Awgu et al., 2011 compared participants’ perspectives on buprenorphine versus methadone in a jail treatment setting [14]. The study concluded that the participants were more prone to receive buprenorphine treatment because of alleviated cravings and a lack of side effects. In addition, the study participants emphasized that they would consider recommending this treatment to other inmates. Similarly, Brinkley-Rubinstein et al., 2019 conducted interviews with incarcerated participants who had access to all three medications approved by the Food and Drug Administration to treat opioid use disorder: methadone, buprenorphine, and naltrexone [15]. The study participants reported different advantages of receiving MAT treatment, including diminished withdrawal symptoms and reduced usage of illicit drugs while incarcerated.

### 3.2. Facilitators and Barriers to Treatment Programs in the Criminal Justice System

Three studies explored the obstacles that hinder access to MAT programs in the US criminal justice system and presented avenues to improve treatment programs and facilitate care for the incarcerated participants. For example, Hanna et al., 2019 explored the perceptions of providers, policymakers, stakeholders, and facility staff to develop programs for incarcerated participants that address their needs regarding MAT access [16]. The study highlighted the opportunity to increase access to MAT education materials and how to navigate the system to decrease the mental health burden. Matusow et al., 2013 surveyed persons incarcerated, and the last open-ended question collected the participants’ views on MAT programs in drug courts [17]. Some of the responses mentioned the possibility of diversion of the medication intended for treatment and misuse. Mitchell et al., 2021 study examined the benefits of a more manageable and friendlier navigating system that allowed the participants to access the MAT treatment [19]. Patients found comfort when people associated with the treatment programs provided them with opportunities to obtain other wrap around services helping with such necessities as food security (eg., food stamps) and assistance regarding navigating the criminal justice system. Contact with patients that remained consistent throughout treatment was commonly reported by those adherent participants of the MAT programs.

## 4. Discussion

Of 6709 unique abstract results identified, there were seven qualitative studies that met the inclusion criteria for this study. This is the first qualitative systematic literature review conducted in the US criminal justice system to capture access to SUD care. Two themes were identified from these results: (1) managing withdrawal from medication-assisted treatment, and (2) facilitators and barriers to treatment programs in the criminal justice system. Qualitative research evaluating access to SUD care in the US criminal justice system varied widely, with some interventions reported not rooted in evidence-based medicine. An opportunity may exist to develop best practices to ensure evidence-based treatment for SUDs is delivered to persons in the US criminal justice system.

The first theme we identified highlighted the existence of a non-evidence-based treatment approach that still may exist in the US criminal justice system—detoxification, whether via tapering or simply stopping. Delivering evidence-based treatment requires the provider to use the available scientific evidence available to inform their clinical decision-making, with the ultimate purpose of providing the best patient care possible [20]. According to the national clinical practice guidelines for the management of opioid use disorder, which is a compilation and interpretation of the most current scientific research available to date, the gold standard of treatment is combining medication therapy with psychosocial support [21]. Research has demonstrated the rate patient’s return to use of the illicit substance is more common after tapering off their treatment medication [22]. Prior research also shows retention in treatment is greater when patients receive medication (methadone or buprenorphine) plus counseling versus counseling alone [23,24]. The literature also shows that overdose mortality increases when a patient’s treatment medication is stopped [25]. Our systematic literature review highlighted patient reports where their treatment medication was tapered or stopped completely. While there are many complexities that influence whether a person involved with the criminal justice system receives SUD treatment, such as length of jail stay, willingness to accept treatment, environmental issues, and others [26], an opportunity may exist to improve efforts to ensure persons with SUDs in the criminal justice system do receive evidence-based care.

The second theme our results identified the barriers and facilitators to providing access to care in the criminal justice system, notably that education around evidence-based treatment is lacking. There are many myths that are perpetuated about medication treatment to manage opioid use disorder, such as “the medication is replacing one addiction with another [27].” These myths are not rooted in evidence but rather in stereotypes and stigmatizing language that still exist in society today due to lack of understanding of that SUDs are disease and available and effective treatments exist to manage them. Increasing educational efforts of all key stakeholders, including persons involved with, providers employed by, and other key personnel in the criminal justice system, may serve as a starting point to help patients seek the care they need. These educational efforts, at a minimum, should include general information about the pathophysiology of the disease and the available and effective treatment options as well as specific access and care navigation information for their facility. While resources exist for providers in these settings, additional resources focused on educating the individual may be needed [28] More research is needed to optimize the delivery of educational programs specifically tailored to the individual engaged with the criminal justice system to achieve maximum benefit.

The issue of access to evidence-based care in the criminal justice system is not new; however, innovative approaches to ensure patients receive or are maintained on their treatment regimen are lacking. Qualitative research provides robust data to better understand the person (and sometimes the patient) perspective about access to evidence-based treatment in the criminal justice system. This study highlights the apparent lack of research on MAT treatment in prisons. This is important because the patient is a key stakeholder in their own care and treatment should be a shared decision between provider and patient [28]. Shared decision making has the ability to impact patient care positively or negatively. For example, it can increase the trust between doctors and patients as it has in Swedish intensive care units or it can cause a disconnect between how nurses and patients understand patient dignity as it did in Iran [29,30]. Shared decision making is a critical component of evidence-based treatment.

## 5. Limitations

As with any study, this study has some limitations. First, our results lacked regional diversity. Of the seven qualitative studies, most took place in the northeastern part of the US. While Matusow et al., 2013 gathered data from 47 of the 50 states and Washington D.C., the study did not stratify their findings by region, and therefore fails to inform about how the answers they received differ by geographical region [17]. Second, non-peer-reviewed articles, conference abstracts, commentaries, discussion articles, court cases, civil-society issues, and others, were excluded which may have limited the breadth of our findings. Third, some publications could have been missed because they might not have been correctly indexed in the databases at the time of the search.

## 6. Conclusions

Seven qualitative studies were included in the systematic literature review, and two themes were identified: (1) managing withdrawal from medication-assisted treatment and (2) facilitators and barriers to treatment programs in the criminal justice system. Qualitative research evaluating access to SUD care in the US criminal justice system varied widely, with some interventions not rooted in evidence-based medicine. An opportunity may exist to develop best practices to ensure evidence-based treatment for SUDs is delivered to persons in the US criminal justice system, such as providing treatment in accordance with accepted clinical practice guidelines and educating individuals on the benefits of that treatment.

## Figures and Tables

**Table 1:** Analysis of Eligible Qualitative Studies (n = 7).

Author and Year of Publication	Population	Methods: Interview (I) Focus Group (FG) Both: I and FG	Treatment: MAT: Methadone (M) Buprenorphine (B) Naltrexone (N)	Withdrawal	Limited Withdrawal	Facilitators and Barriers to MAT Programs	Locations	Number of Participants	Eligibility Criteria
Aronowitz et al., 2016 [13]	Formerly Incarcerated	I	M, B before incarceration	*			Methadone and buprenorphine outpatient treatment centers in northern New England	10	Incarceration, forced MAT taper, English speaker, 18 years or older
Awgu et al., 2011 [14]	Incarcerated	I	M, B	*			Key Extended Entry Program (KEEP) within the Rikers Island jail complex in New York City	133	Opioid use disorder and a sentence less than 1 year long
Brinkley-Rubinstein et al., 2019 [15]	Incarcerated	I	M, B, N	*	*		Rhode Island Department of Corrections MAT	40	MAT, 18 or older, able to speak and write in English
Hanna et al., 2019 [16]	Stakeholders, providers, facility staff, policymakers	Both					Michigan Department of Corrections (MDOC)	Not reported	Staff and participants at at Michigan department of correction MAT
Matusow et al., 2013 [17]	People involved in drug courts	Neither: open ended question					47 states plus D.C and Puerto Rico	103 responses	Surveys sent to drug courts in 49 states plus D.C and Puerto Rico
Mitchell et al., 2011 [18]	methadone maintenance program participant	I	M	*		*	Baltimore Maryland	92	In treatment group found at Baltimore methadone clinics. Out of treatment found through targeted sampling
Mitchell et al. 2021 [19]	Incarcerated and post-release	I	M			*	Baltimore Maryland	17	Methadone treatment program in Baltimore receiving IM + PN services

The * means the information was presented.

## Data Availability

Not applicable.

## Appendix A. Appendix of Strategies

### Appendix A.1. PubMed/MEDLINE Search Strategy

(((“health services accessibility” OR “patient acceptance of health care” OR “health knowledge” OR “health attitudes” OR “health practices” OR “right to health” OR “treatment refusal” OR “health equity” OR “health services accessibility”[mesh] OR “patient acceptance of health care”[mesh] OR “health knowledge, attitudes, practice”[mesh] OR “right to health”[mesh] OR “treatment refusal”[mesh] OR “health equity”[mesh] OR “health policy”[mesh] OR “ethics, clinical”[mesh] OR “Public Health/drug therapy”[Mesh] OR “Public Health/education”[Mesh] OR “Public Health/ethics”[Mesh] OR “Public Health/rehabilitation”[Mesh])) AND ((prison*[tiab] OR prisoner[tiab] OR criminal*[tiab] OR incarcerat*[tiab] OR jail*[tiab] OR penitentiar*[tiab] OR crime[tiab] OR legal*[tiab] OR “law enforcement”[tiab] OR “criminal law”[tiab] OR “correctional facility”[tiab] OR “justice system”[tiab] OR “crime”[mesh] OR “prisoners”[mesh] OR “jails”[mesh] OR “prisons”[mesh] OR “correctional facilities”[mesh] OR “criminal law”[mesh] OR “law enforcement”[mesh]))) AND ((opioid[tiab] OR heroin[tiab] OR methamphetamine[tiab] OR cocaine[tiab] OR MDMA[tiab] OR ecstasy[tiab] OR N-Methyl-3,4-methylenedioxyamphetamine[tiab] OR fentanyl[tiab] OR narcotic[tiab] OR substance abuse OR drug abuse OR illicit drugs OR party drugs OR street drugs OR “heroin dependence”[mesh] OR “opiate substitution treatment”[mesh] OR “opioid-related disorders”[mesh] OR “illicit drugs”[mesh] OR “substance-related disorders”[mesh] OR “substance abuse treatment centers”[mesh] OR “amphetamine-related disorders”[mesh] OR “cocaine-related disorders”[mesh] OR “narcotic-related disorders”[mesh] OR “cocaine”[mesh] OR “cocaine smoking”[mesh] OR “methamphetamine”[mesh] OR “N-Methyl-3,4-methylenedioxyamphetamine”[mesh] OR “fentanyl”[mesh])).

### Appendix A.2. Embase Search Strategy

Line 1

‘health services accessibility’:ti,ab OR ‘patient acceptance of health care’:ti,ab OR ‘health knowledge’:ti,ab OR ‘health attitudes’:ti,ab OR ‘health practices’:ti,ab OR ‘right to health’:ti,ab OR ‘treatment refusal’:ti,ab OR ‘health equity’:ti,ab OR ‘health policy’:ti,ab OR ‘clinical ethics’:ti,ab OR ‘public health’:ti,ab OR ‘health service’/exp OR ‘attitude to health’/exp OR ‘patient attitude’/exp OR ‘right to health’/exp OR ‘treatment refusal’/exp OR ‘health equity’/exp OR ‘health care policy’/exp OR ‘public health’/exp.

Line 2

‘prison*’:ti,ab OR ‘prisoner*’:ti,ab OR ‘criminal*’:ti,ab OR ‘incarcerat*’:ti,ab OR ‘jail*’:ti,ab OR ‘penitentiar*’:ti,ab OR ‘crime’:ti,ab OR ‘legal*’:ti,ab OR ‘law enforcement’:ti,ab OR ‘criminal law’:ti,ab OR ‘correctional facility’:ti,ab OR ‘justice system’:ti,ab OR ‘criminal law’/exp OR ‘law enforcement’/exp OR ‘correctional facility’/exp OR ‘crime’/exp OR ‘prisoner’/exp.

Line 3

‘opiate’/exp OR ‘diamorphine’/exp OR ‘methamphetamine’/exp OR ‘cocaine’/exp OR ‘midomafetamine’/exp OR ‘fentanyl’/exp OR ‘narcotic agent’/exp OR ‘substance abuse’/exp OR ‘drug abuse’/exp OR ‘illicit drug’/exp OR ‘street drug’/exp OR ‘drug dependence treatment’/exp OR ‘opioid’:ti,ab OR ‘heroin’:ti,ab OR ‘methamphetamine’:ti,ab OR ‘cocaine’:ti,ab OR ‘mdma’:ti,ab OR ‘ecstasy’:ti,ab OR ‘n methyl 3,4 methylenedioxyamphetamine’:ti,ab OR ‘fentanyl’:ti,ab OR ‘narcotic’:ti,ab OR ‘substance abuse’:ti,ab OR ‘drug abuse’:ti,ab OR ‘illicit drugs’:ti,ab OR ‘party drugs’:ti,ab OR ‘street drugs’:ti,ab.

### Appendix A.3. Scopus Search Strategy

(TITLE-ABS-KEY (prison* OR prisoner* OR criminal* OR incarcerat* OR jail* OR penitentiar* OR crime OR legal* OR “law enforcement” OR “criminal law” OR “correctional facilit*” OR “justice system” OR crime))AND (TITLE-ABS-KEY (“health services accessibility” OR “patient acceptance of health care” OR “health knowledge” OR “health attitudes” OR “health practices” OR “right to health” OR “treatment refusal” OR “health equity” OR “health policy” OR “clinical ethics” OR “public health”)) AND (TITLE-ABS-KEY (opioid OR heroin OR methamphetamine OR cocaine OR mdma OR ecstasy OR n-methyl-3,4-methylenedioxyamphetamine OR fentanyl OR narcotic OR “substance abuse” OR “drug abuse”OR”illicit drugs” OR “party drugs “OR”street drugs”)).
